# Secretory stressors induce intracellular death receptor accumulation to control apoptosis

**DOI:** 10.1038/cddis.2017.466

**Published:** 2017-10-05

**Authors:** Bram J van Raam, Tamara Lacina, Ralph K Lindemann, Jan H Reiling

**Affiliations:** 1 Metabolism and Signaling in Cancer, BioMed X Innovation Center, Im Neuenheimer Feld 583, Heidelberg 69120, Germany; 2 Merck Serono TA Oncology, Merck KGaA, Frankfurter Str. 250, Darmstadt D-64293, Germany

## Abstract

Disruption of the Golgi apparatus can induce a distinct form of programmed cell death that has not been thoroughly characterized. We found that pharmacological application of Golgi stress leads to induction of death receptors (DRs) 4 and 5. DR4 appears to be primarily responsible for the initiation of cell death downstream of Golgi stress, whereas DR5 seems to be more important for cell death triggered by endoplasmic reticulum (ER) stress in specific cancer cell lines. DR induction downstream of either Golgi or ER stress mainly causes intracellular accumulation of DR4 presumably at the Golgi, rather than increased expression on the cell surface. Nevertheless, cells treated with secretory pathway stressors displayed an increased susceptibility to TRAIL (tumor necrosis factor related apoptosis inducing ligand), the endogenous ligand of DR4/5, probably due to intracellular sequestration of the caspase-8 regulator CFLAR (caspase-8 and FADD-like apoptosis regulator). These findings have implications for the treatment of cancer with DR agonists and our general understanding of DR signaling while highlighting the role of the Golgi apparatus as a cell death signaling platform.

The Golgi apparatus is a highly dynamic organelle that, together with the endoplasmic reticulum (ER), is responsible for the distribution of newly synthesized proteins and lipids throughout the cell. Interruption of the vesicle stream from the ER causes a rapid loss of Golgi coherence. It has previously been shown that prolonged, chemically induced, Golgi disruption (or ‘Golgi stress’) induces activation of the transcription factor CREB3 (cyclic AMP-responsive element-binding protein 3; Luman or LZIP) leading to induction of the small GTP-binding protein ADP ribosylation factor 4 (ARF4) and cell death.^1^ Golgi stress can be triggered by several compounds, including the protein secretion inhibitors brefeldin A (BFA) and golgicide A (GCA), which both trap a subset of complexes formed between the ARFs and some of their guanine nucleotide exchange factors in an unproductive conformation.^2, 3^ Other compounds known to affect Golgi structure and activate the Golgi stress program are AG1478^4^ (tyrphostin), which displays a similar mode of action to BFA and GCA, and monensin (MNS), an ionophore for monovalent cations.^5^

ER stress is commonly induced by compounds such as tunicamycin (TUN), an inhibitor of *N*-linked glycosylation, or thapsigargin (THA), an inhibitor of the ER-specific Ca^2+^ transporter SERCA (sarco/endoplasmic reticulum Ca2^+^-ATPase). ER stress signaling is mediated through the evolutionarily conserved unfolded protein response (UPR). Accumulation of unfolded proteins in the ER lumen leads to dissociation of chaperone proteins such as GRP78 (glucose-regulated protein 78; BiP/HSPA5) from several transmembrane proteins in the ER including PKR-like ER kinase (PERK), activating transcription factor 6 (ATF6) and inositol-requiring enzyme 1*α* (IRE1*α*), leading to their activation and the initiation of the ER stress response.^6^ Initially, ATF6 and PERK activation activate a rescue program to facilitate return to homeostasis.^7^ However, prolonged ER stress eventually triggers a cell death program about which some controversies still exist.^8^ Importantly, ER stress leads to the induction of the transcription factor CHOP (C/EBP-homologous protein) downstream of ATF6 and PERK signaling. CHOP induces the expression of the cell surface death receptors 4 and 5 (DR4/5)^9, 10^ and other cell death promoting genes, while reducing the expression of anti-apoptotic genes.^7, 8, 11^ DR5 induction sensitizes cells to TRAIL (tumor necrosis factor related apoptosis inducing ligand), the endogenous ligand for both DR5 and DR4.^12, 13, 14^ Intracellular aggregation of DR5 during ER stress can lead to ligand-independent signaling through the receptor.^15^ More recently, another group has claimed that DR4, rather than DR5, is the main inducer of cell death downstream of ER stress.^16^

DR4/5 are members of the tumor necrosis factor receptor super family (TNFRSF). Most of the receptors in this family do not trigger cell death directly, but only when the expression of anti-apoptotic and pro-survival proteins is prevented.^17^ However, DR4/5 can both directly interact with the adaptor protein FADD (FAS-associated death domain protein) through their intracellular death domains. FADD, in turn, recruits and activates caspase-8 through forced dimerization via its death effector domain.^18^ Active caspase-8 then proceeds to cleave downstream apoptotic substrates.^19^

In the current study, we address the controversy surrounding the involvement of DR4 or DR5 in the initiation of cell death downstream of secretory stress. In addition, we further elucidate the nature of programmed cell death triggered by prolonged Golgi disruption and the factors involved therein. We uncover that DR4 and DR5 play distinct roles in the initiation of apoptotic cell death downstream of Golgi stress or ER stress, respectively.

## Results

### Golgi stress leads to the specific induction of DR4 and DR5 expression

**Figure 1:**
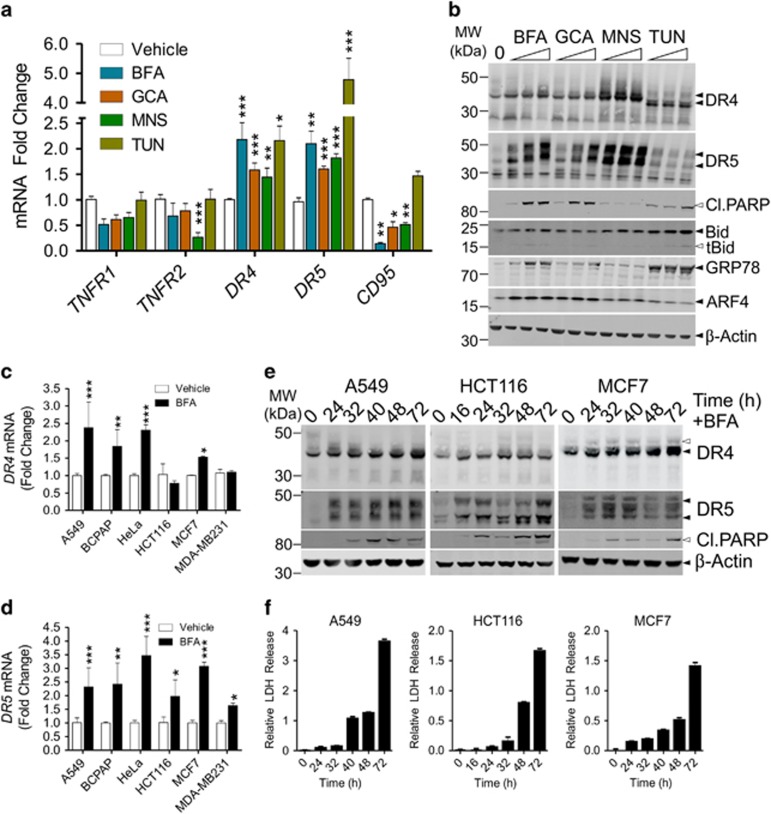
Induction of death receptors 4 and 5 upon application of Golgi stress. (**a**) A549 cells were incubated with vehicle (EtOH), BFA (100 nM), GCA (1 *μ*M), MNS (5 *μ*M) or tunicamycin (TUN; 10 *μ*M) for 24 h after which mRNA was isolated and cDNA prepared. Relative expression of the indicated TNF-receptor superfamily members was determined by qPCR. The fold change relative to the vehicle-treated control is shown. Values represent the mean±S.D. of three independent experiments with duplicate technical replicates. A two-way ANOVA was performed to determine whether the expression levels found in the treated samples were significantly different from the vehicle controls. *P<0.05, **P<0.01,***P<0.001. (**b**) A549 cells were incubated for 48 h with increasing concentrations of BFA (40, 60 or 100 nM), GCA (2, 2.5 or 3 *μ*M), MNS (5, 10, 20 *μ*M), TUN (20, 40, 80 *μ*M) or vehicle (EtOH; 0), after which samples were prepared for western blot. Blots were probed with specific antibodies against the indicated proteins and re-probed for *β*-Actin as a loading control. Black arrowheads indicate full-length proteins and white arrowheads their cleavage products. DR4 and DR5 are glycosylated proteins, resulting in a visible smear on western blot. Upon treatment with TUN, glycosylation is prevented and DR4 in particular appears at a slightly lower molecular weight on the blot. The two main isoforms of DR5 (short and long) are also indicated. Blots are representative of three independent experiments. (**c,d**) Different cancer cell lines were incubated for 24 h with either vehicle (EtOH) or 100 nM BFA. Afterwards, mRNA was isolated and cDNA prepared to determine the relative induction of *DR4* and *DR5* by RT-PCR. Data represent the mean±S.D. of triplicate experiments. *P<0.05, **P<0.01,***P<0.001, relative to the vehicle control (two-way ANOVA). (**e**,**f**) A549 (left), HCT116 (middle) or MCF7 cells (right) were incubated with either 100 nM, 80 nM or 200 nM BFA, respectively, to induce a robust cell death response. Lysates were prepared for western blot (**e**) while samples of the culture supernatant were taken for LDH release analysis as a relative measure of cell death (**f**). Blots were probed with specific antibodies against cleaved PARP, DR4, DR5 and re-probed for *β*-Actin as a loading control. Black arrowheads indicate full-length proteins and their splice variants, white arrowheads cleavage products. Representative LDH release data ((treated/untreated)-1) is shown as the mean±S.D. of triplicate samples

The expression of relevant members of the TNFRSF following secretory stress was investigated by semi-quantitative real-time PCR (qPCR) in both the lung cancer cell line A549 (Figure 1a) and the thyroid cancer cell line BCPAP (Supplementary Figure S1a). In line with previous observations using the colon cancer cell line HCT116,^15^ we found significant upregulation of *DR5* (*TNFRSF10B*) after the application of secretory stress (Figure 1a and Supplementary Figures S1a–c). We also observed consistent upregulation of *DR4* (*TNFRSF10A*) in both cell lines. At the protein level, induction of both DRs could be observed in A549 cells treated with different secretory stressors (Figure 1b). DR4/5 induction occurred with relatively low doses of the stimuli and preceded significant apoptotic cell death, as indicated by cleavage of the downstream caspase substrate PARP (poly [ADP-ribose] polymerase 1) and the caspase-8 substrate Bid. The Golgi stress marker ARF4 was induced by relatively low doses of the Golgi stressors, but not by TUN, whereas TUN readily caused upregulation of the ER stress marker GRP78, which was only robustly induced by high doses of the Golgi stressors. A panel of additional cancer cell lines was tested for the induction of *DR4* and *DR5* at the mRNA level upon Golgi stress treatment (Figures 1c and d). HeLa (cervical cancer) and MCF7 (breast cancer) cells also displayed enhanced expression of both *DR4* and *DR5* in response to BFA, whereas HCT116 and MDA-MB231 (breast cancer) cells only showed significant upregulation of *DR5* mRNA.

At the protein level, A549, HCT116 and MCF7 cells displayed induction of both DR4 and DR5 upon BFA treatment (Figure 1e). DR induction preceded the significant onset of cell death, indicated by PARP cleavage and lactate dehydrogenase (LDH) release from the cells as an indicator of late apoptosis/necrosis (Figure 1f). Thus, induction of both DR4 and DR5 was consistently observed by western blot analysis, although significant *DR4* mRNA induction was not observed in HCT116 cells. This might suggest a different mode of regulation in these cells or a difference in dynamics.

### DR4 is involved in the initiation of Golgi-stress-induced cell death

**Figure 2:**
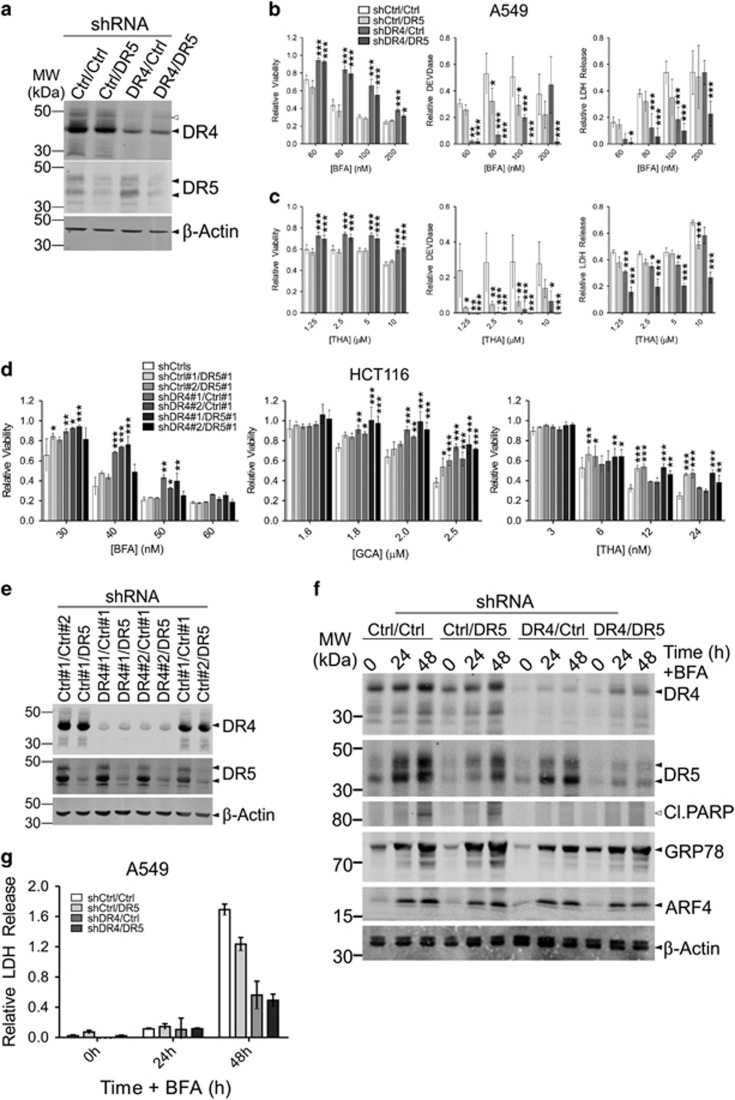
Knockdown of DR4 protects cells from Golgi-stress-induced cell death. (**a**–**e**) A549 cells (**a**–**c**) and HCT116 cells (**d**,**e**) stably transduced with two independent shRNA constructs with different antibiotic resistance genes either targeting control genes (Ctrl; *Luciferase* (#1) or *GFP* (#2)), *DR4* or *DR5* in different combinations were tested for their sensitivity to BFA, GCA or thapsigargin (THA). Knockdown was confirmed by western blot analysis (**a** for A549 cells and **e** for HCT116 cells), probed for either DR4 or DR5 and re-probed for *β*-Actin as a loading control. Relative cell viability, assessed with a CTB assay, caspase-3/7 activity (DEVDase), and relative LDH release in the culture supernatant were determined 48 h after the addition of increasing concentrations of the indicated compounds (**b**,**c**) for A549 cells and (**d**) for HCT116 cells. Relative values (treated/untreated (viability) or ((treated/untreated)−1) (DEVDase and LDH release)) represent the mean±S.D. of three independent experiments performed in triplicate for each cell line/condition. A two-way ANOVA was performed to determine whether the relative values obtained for the experimental KD cells was significantly different from the control KDs. *P<0.05, **P<0.01,*** *P*<0.001. (**f**,**g**) A549 cells depleted of DR4, DR5 or both, as above, were incubated with 100 nM BFA for up to 48 h. Samples were taken from the cell culture supernatant to determine LDH release as a relative measure of cell death (**f**). Relative values ((treated/untreated)−1) represent the mean±S.D. of triplicate samples. Cell lysates were prepared at the indicated time points and analyzed on western blot (**g**). Blots were probed for the indicated proteins and re-probed for *β*-Actin as a loading control. Black arrowheads indicate full-length proteins and their splice variants, white arrowheads their cleavage products, where appropriate. Blots are representative of three independent experiments

Knockdown (KD) cells for either DR individually or both DRs together (DKD) were generated by stably transducing different cell lines with specific shRNA constructs targeting one or both of these DRs as well as control genes (*Luciferase* (Ctrl#1) or *GFP* (Ctrl#2)). Cells were tested for their susceptibility to different compounds using the CellTiter-Blue (CTB) assay to determine relative viability in combination with a DEVDase assay to determine activation of caspase-3/7 as an indicator of apoptotic cell death, and an LDH release assay to determine late apoptosis/necrosis. DR4 KD or DR4/5 DKD A549 cells, but not DR5 KD cells, displayed clear resistance to BFA and THA on the viability level (Figures 2a–c and Supplementary Figure S2a). However, DEVDase activity was also reduced in the DR5 KD cells treated with THA, and the DR4/5 DKD cells treated with either BFA or THA displayed a greater reduction in LDH release than the single DR4 or DR5 KD cells. This indicates that both DR4 and DR5 play a role in secretory-stress-induced cell death, but may differ in their ability to induce apoptosis or reduce cell growth. DR4 KD HCT116 cells were similarly resistant to BFA and GCA, but only DR5 KD HCT116 cells displayed resistance to THA (Figures 2d–e and Supplementary Figure S2b). Noticeable differences could be observed between the response to BFA and the response to THA in the dose-response curves of the different KD cell lines (Supplementary Figures S2a and b). The curves of BFA-resistant cells displayed a right-shift, indicating that a greater dose of BFA is required to elicit a response from these cells, though the cells displayed the same extent of cell death as the controls at higher doses of BFA. This suggests that other cell death mechanisms besides DR activation are also engaged. Cell lines resistant to THA displayed an increased drug ceiling indicating significant resistance to the compound. This suggests that these compounds activate both common and unique cell death pathways. When HeLa cells deficient in either DR4 or DR5 were generated, cells stably transduced with DR5 shRNA constructs displayed increased expression of DR4 (Supplementary Figures S2c and d). Subsequently, while the DR4 KD HeLa cells were resistant to treatment (Supplementary Figure S2e), the cells transduced with DR5 shRNAs were actually sensitized (Supplementary Figure S2f), presumably due to the increased expression of DR4 in these cells. Note that neither DR4- nor DR5-deficient cells were resistant to monensin (MNS; Supplementary Figure S2f), even though this compound induces the expression of DR4/5. This indicates that induction of DRs does not necessarily lead to their activation, and not all compounds that induce secretory stress induce DR-dependent cell death.

Finally, A549 DKD cells were treated with BFA for western blot analysis (Figure 2f). This revealed a reduced induction of GRP78 in the single DR4 and DR4/DR5 DKD cells, suggesting diminished ER stress, while ARF4 induction occurred normally. In addition, DR5 induction in DR4 KD cells appeared to be slightly reduced following BFA treatment as compared to the control. Together, these data suggest that DR4 signaling and cell death initiation is triggered by Golgi stress, which eventually leads to ER stress and DR5 induction, resulting in amplification of cell death signaling.

### Induction of DR4/5 is partially dependent on CHOP

**Figure 3:**
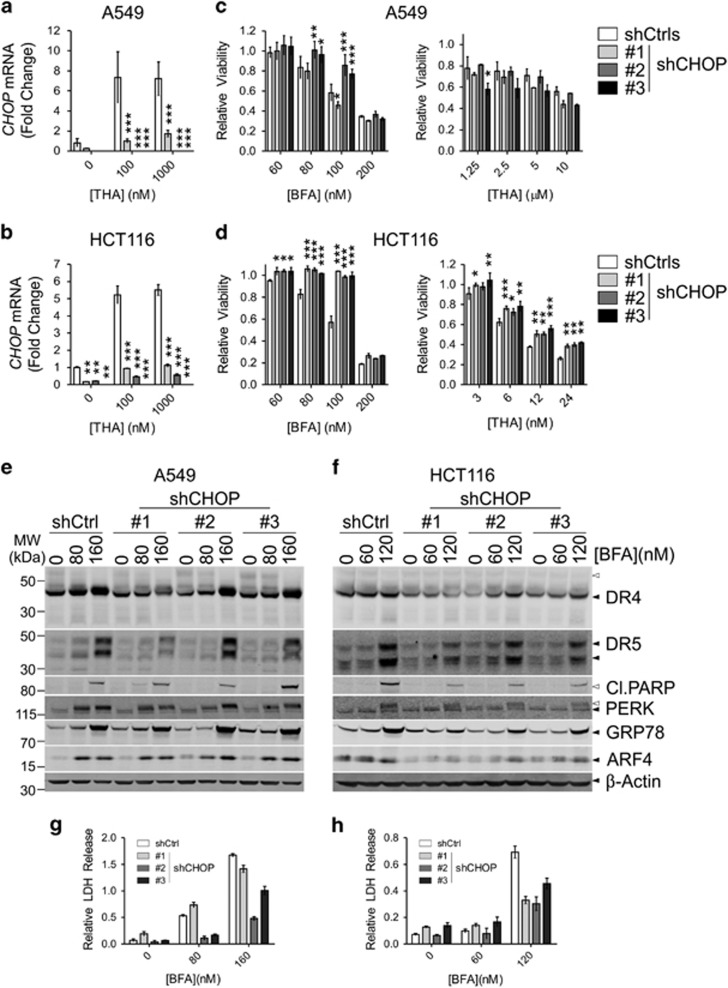
Knockdown of *CHOP* protects cells from secretory stress. (**a**–**d**) A549 cells (**a**,**c**) and HCT116 cells (**b**,**d**) stably transduced with shRNA constructs targeting control genes (Ctrls; Luciferase and *GFP*) or *CHOP* were tested for their sensitivity to BFA or thapsigargin (THA). Efficient KD of *CHOP* was verified by qPCR in the presence (overnight stimulation) or absence of THA (**a**,**b**), as indicated. Relative cell viability was assessed with a CTB assay 48 h after the addition of different concentrations of the indicated compounds (**c** for A549 cells and **d** for HCT116 cells). Relative values (treated/untreated) represent the mean±S.D. of three independent experiments performed in triplicate for each cell line/condition. A two-way ANOVA was performed to determine whether the expression of *CHOP* or the relative viability of the experimental KD was significantly different from the control KD. *P<0.05, **P<0.01,***P<0.001. (**e**–**h**): A549 cells (**e**,**g**) and HCT116 cells (**f**,**h**) stably transfected with different shRNAs targeting either *CHOP* or a control gene (*Luciferase*; Ctrl) were treated for 48 h with the indicated doses of BFA or vehicle (EtOH). Afterwards, lysates were prepared for western blot (**e**,**f**) and samples were taken of the culture supernatant to determine relative LDH release as a measure of cell death (**g**,**h**). Relative values ((treated/untreated)−1) represent the mean±S.D. of triplicate samples. Blots were probed for the indicated proteins and re-probed for *β*-Actin as a loading control. Black arrowheads indicate full-length proteins and their splice variants, white arrowheads their cleavage products or modified species of the protein. Blots are representative of three independent experiments

A549 and HCT116 cells were transduced with different shRNA constructs targeting either *CHOP* or control genes (Ctrls; *Luciferase* or *RFP*). To demonstrate CHOP knockdown, cells were stimulated with increasing doses of THA followed by qPCR analysis (Figures 3a and b).

CHOP KD cells were treated with increasing concentrations of BFA or THA, after which relative viability was determined with CTB (Figures 3c and d). CHOP depletion protected most cell lines from BFA-induced cell death, except for A549 cells infected with shRNA#1, which still expressed residual levels of CHOP. On the other hand, CHOP KD failed to protect A549 cells significantly from THA treatment. Compared to control cells, CHOP-depleted HCT116 cells were protected from BFA or THA treatment. However, at higher concentrations of either compound, CHOP KD cells still failed to survive suggesting the parallel activation of alternative, pro-death signaling pathways. For example, we identified a common MYC-associated factor X (MAX) transcription factor binding motif (GGACCAAGTGGCAA) in the promoter regions of *DR4*, *DR5* and *ARF4* (see Material and Methods). MAX is a binding partner of c-Myc,^20^ and we found that co-treatment of BFA and 10058-F4, a small molecule inhibitor that prevents c-Myc/MAX heterodimerization^21^ also leads to reduced DR4/5 expression and cell death. Similarly, ARF4 at earlier time points was induced to a lesser extent in cells co-treated with the c-Myc/MAX inhibitor compared to BFA-only treatment (Supplementary Figure S3). This suggests that ARF4 might be a direct c-Myc target, which is in line with a previous report.^22^

Induction of DR4/5 was investigated in the CHOP KD cells by treating them with BFA, followed by western blot and LDH release analysis (Figures 3e–h). DR4/DR5 upregulation was generally reduced in the CHOP-depleted cells as compared to the controls, especially at lower concentrations of BFA. CHOP KD HCT116 cells, but not A549 cells, also displayed reduced ARF4 and GRP78 induction, whereas PERK appeared to be phosphorylated normally as judged by a similar band-shift by immunoblotting in control and CHOP KD cells. This suggests a role for CHOP in the amplification of the stress signal in HCT116 cells. Higher levels of BFA still induced DR4/DR5 expression and death in all cell lines.

**Figure 4:**
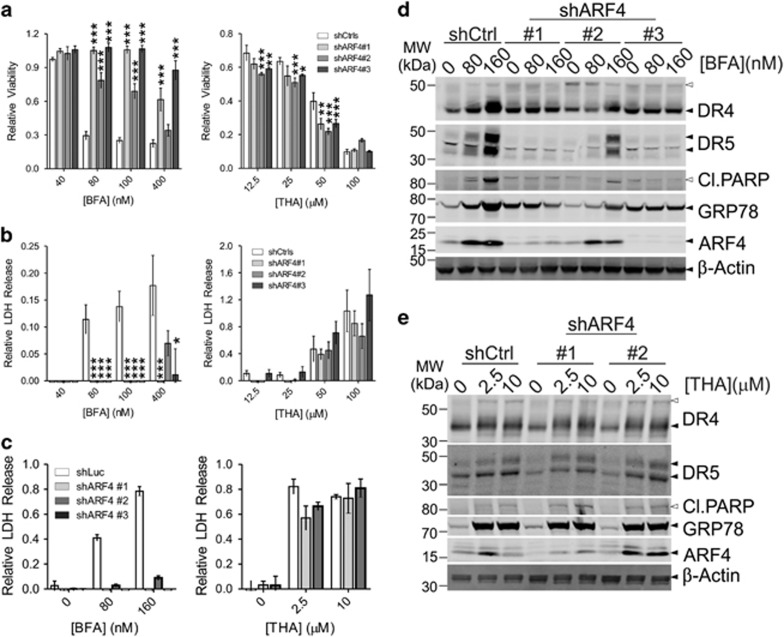
ARF4 knockdown protects from Golgi-stress-induced cell death and induction of DR4/5. (**a**,**b**) A549 cells were stably transduced with different shRNA constructs targeting either control genes (Ctrls; *GFP* or *Luciferase*) or *ARF4* and tested for their sensitivity to either BFA (left panels) or thapsigargin (THA; right panels) by treating the cells for 48 h with increasing concentrations of these compounds. Afterwards, their relative viability was determined by a CTB assay (**a**), while LDH release in the culture supernatant was used as a relative measure of cell death (**b**). Relative values ((treated/untreated) for CTB, ((treated/untreated)−1) for LDH release) represent the mean±S.D. of three independent experiments performed in triplicate for each cell line/condition. A two-way ANOVA was performed to determine whether response of the experimental KDs was significantly different from the control KDs. *P<0.05, **P<0.01,***P<0.001. (**c**–**e**) To determine the induction of DR4 and DR5, control and ARF4 KD A549 cells were treated with vehicle (EtOH; 0) or increasing concentrations of either BFA or thapsigargin (THA). LDH release in the culture supernatant was determined after 48 h as a relative measure of cell death (**c**) and samples were prepared for western blot analysis to determine the expression levels of the indicated proteins (**d**,**e**). Blots were re-probed for *β*-Actin as a loading control. Black arrowheads indicate full-length proteins and their splice variants, white arrowheads their cleavage products or modified species of the protein. Blots are representative of three independent experiments

To investigate whether the observed induction of DR4 and DR5 occurred upstream or downstream of Golgi stress, A549 and HeLa cells were transduced with shRNA constructs targeting either components of the trafficking protein particle complex (TRAPPC), ARF1 or ARF4. TRAPPC11, TRAPPC12, TRAPPC13^23^ and ARF4-depleted^1^ cells are resistant to Golgi stress, but display only limited protection from ER stress (Figure 4 and Supplementary Figure S4). On the other hand, ARF1 KD sensitizes cells to Golgi stress.^1^ The resistance and sensitization phenotypes can also be deduced from the observed PARP cleavage and the induction pattern of ARF4 and the concomitant reduction of ARF1 as well as the (lack of) induction of GRP78 on western blot (Figure 4d and Supplementary Figure S4a). Importantly, induction of DR4 and DR5 was reduced in the BFA-resistant ARF4 or TRAPPC KD cell lines. Thus, DR4/5 upregulation appears to occur as a consequence of Golgi stress following BFA treatment. No significant differences were observed when the cells were treated with THA or TUN (Figure 4e and Supplementary Figure S4b).

### Secretory stress results in intracellular accumulation of DR4 and DR5

ER stress can lead to increased sensitivity of cancer cells to TRAIL treatment due to upregulation of DR5.^24, 25^ Conversely, Lu *et al.* noted that treatment with a high dose (>1 *μ*M) of BFA mostly led to intracellular clustering of DR5 and therefore did not sensitize cells to TRAIL treatment.^15^

**Figure 5:**
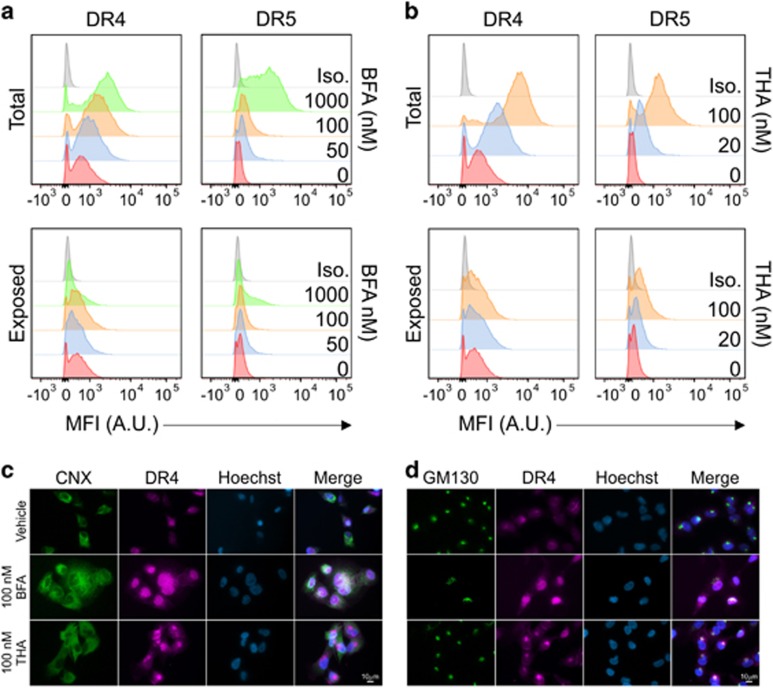
Secretory stress leads to intracellular accumulation of DR4. (**a**,**b**) A549 cells were treated for 24 h with increasing concentrations of BFA (**a**) or thapsigargin (THA) (**b**). Afterwards, the cells were collected, fixed and divided over different samples. Half the samples were permeabilized (top) before probing with specific antibodies against either DR4 (left) or DR5 (right) to determine total (intracellular and membrane) expression of the DRs, the other half was left unpermeablized to determine the fraction of DRs exposed on the cell membrane (bottom). Data were collected by FACS analysis and representative histograms of three independent experiments are shown, depicting cell count over mean fluorescence intensity (MFI) expressed in arbitrary units (A.U.) as compared to staining with the isotype control antibody (Iso.). (**c**,**d**) A549 cells were grown on microscopy cover slips and incubated with either vehicle (EtOH), 100 nM BFA or 100 nM thapsigargin (THA) for 24 h. Afterwards, the cells were washed, fixed, permeabilized and probed overnight with specific antibodies against either the ER marker Calnexin (CNX; **c**) or the Golgi marker GM130 (**d**) in combination with an antibody against DR4. The following day, cells were washed again and probed with fluorescently labeled secondary antibodies to detect the localization of DR4 as well as Hoechst to stain the nuclei. Coverslips were then mounted on slides and analyzed by fluorescence microscopy. Representative images of single channels and overlays are shown

To investigate DR4 localization, A549 cells were treated with increasing doses of BFA or THA. DR expression was analyzed by flow cytometry in permeabilized and in intact cells to detect either total or surface-exposed DR expression, respectively (Figures 5a and b). A dose-dependent increase in total, but not plasma membrane-exposed, DR4/5 expression was observed upon treatment. Cell surface expression of DR5 increased only marginally. The highest dose of BFA led to a relative decrease of receptor exposure, in line with a previous report,^15^ reflecting a severe disruption of protein secretion in these cells.

DR5 has previously been suggested to localize at the Golgi apparatus upon THA treatment.^15^ To determine the localization of DR4, A549 cells were either left untreated or treated with THA or BFA. Cells were stained for DR4 in combination with either calnexin (CNX) as a marker for the ER or GM130 as a marker for the Golgi apparatus. Upon analysis by fluorescence microscopy, it became apparent that treatment with THA in particular resulted in perinuclear localization of DR4, overlapping with GM130 staining, but not with CNX (Figures 5c and d and Supplementary Figures S5a and b). Exposure of cells to BFA leads to partial fusion of the Golgi with the ER thereby creating a hybrid compartment.^26^ Accordingly, DR4 staining became more diffuse, though appeared to be concentrated in the general area of the dispersed Golgi in agreement with a previous report.^27^ Thus, it appears that application of secretory stress generally leads to DR accumulation in the Golgi apparatus, suggesting that this organelle forms a platform for DR signaling in stressed cells.

### Secretory stress sensitizes cancer cells to TRAIL treatment

**Figure 6:**
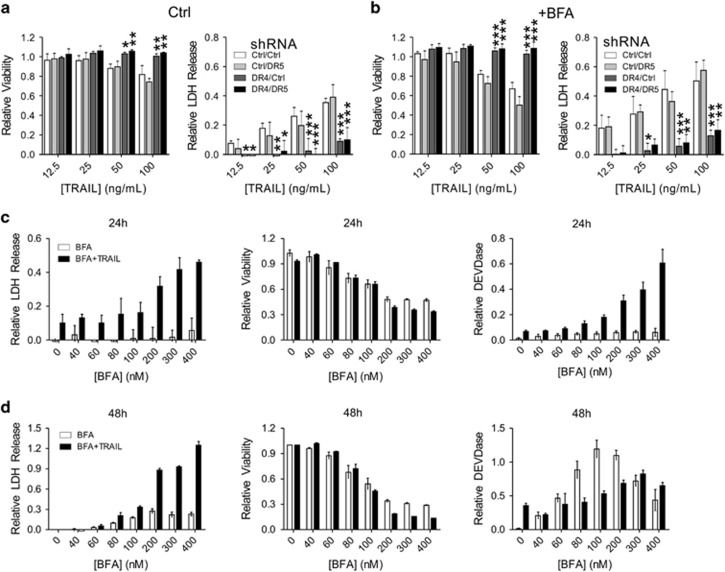
Induced expression of DR4 by Golgi stress sensitizes A549 cells to TRAIL. (**a**,**b**) A549 cells stably transduced with specific shRNA constructs targeting either a control gene (*Luciferase*), *DR4*, *DR5* or both were exposed to increasing concentrations of TRAIL alone (**a**) or TRAIL plus 30 nM BFA (**b**) for 48 h. TRAIL was added 6 h after the addition of BFA or medium. Afterwards, relative cell viability was determined with a CTB assay (left) while LDH release in the cell culture supernatant was determined as a relative measure of cell death (right). Relative values (treated/untreated; CTB) or ((treated/untreated)−1; LDH release) represent the mean±S.D. of three independent experiments performed in triplicate for each cell line/condition. A two-way ANOVA was performed to determine whether the phenotype of the experimental KD cells was significantly different from the control KD upon treatment. *P<0.05, **P<0.01,***P<0.001. (**c**,**d**) A549 cells were incubated with increasing concentrations of BFA in the presence (black bars) or absence (white bars) of 25 ng/ml TRAIL for either 24 h (**c**) or 48 h (**d**). TRAIL was added 6 h after the addition of BFA or medium. Afterwards, LDH release in the cell culture supernatant was determined as a relative measure of cell death (left), relative cell viability was determined with a CTB assay (middle) while DEVDase activity was determined as a relative measure of caspase-3/7 activity, indicative of apoptotic cell death. Relative values (treated/untreated; CTB) or ((treated/untreated)−1; LDH release and DEVDase) represent the mean±S.D. of triplicate experiments

Even though treatment with low doses of BFA did not lead to a marked increase in DR4/5 cell surface expression, we nevertheless decided to investigate whether this could sensitize TRAIL-resistant A549 cells to TRAIL.^28^ Control, DR4 KD, DR5 KD or DR4/5 DKD A549 cells (Figure 2) were either left untreated or treated with a sub-lethal dose of BFA, followed by treatment with increasing doses of TRAIL, after which relative viability and LDH release were determined. Only the DR4-depleted cells displayed an increased resistance to TRAIL, indicating that DR4 is the main TRAIL receptor for these cells (Figure 6a). While pre-treatment with BFA sensitized both control and DR5-deficient cells to TRAIL, the DR4- and DR4/DR5-deficient cells were resistant to the combination treatment as well (Figure 6b). Thus, intracellular accumulation of DR4 in WT or DR5-depleted cells can still lead to increased sensitivity of cancer cells to TRAIL.

HCT116 cells are very susceptible to TRAIL. It was previously suggested that these cells preferentially use DR5 to transmit the TRAIL signal.^29^ To investigate whether this might explain their susceptibility, TRAIL was titrated on control, DR4 KD, DR5 KD and DR4/5 DKD HCT116 cells (Supplementary Figures S6a and b). Only DR4-depleted HCT116 cells displayed resistance to TRAIL. This suggests that DR4 is the main death-inducing receptor for TRAIL whereas DR5, which is more strongly induced during stress conditions, may only act as the main TRAIL receptor under particular conditions in certain cell types. To investigate this in more detail, several Golgi or ER stress-inducing agents were titrated on A549 cells with either plain medium or medium containing 25 ng/ml TRAIL added 6 h after the secretory stressors (Figures 6c and d and Supplementary Figures S6c–g). In all cases, cells treated with TRAIL were more susceptible to secretory stress. This susceptibility was mostly reflected in a relative increase of LDH release, especially after 24 h, indicating an increased rate of apoptosis rather than an increase in the number of affected cells. The DEVDase activity determined for these same cells also reflects this. Relative viability, which is reflective of cell growth and metabolic activity rather than death was not always affected by TRAIL co-treatment especially with the Golgi stressors. This suggests that these compounds may exert an early effect on mitochondria and cellular metabolism affecting the ability of the cells to metabolize resazurin to resorufin in the CTB assay, which is only followed by a full-blown apoptotic response later, except when the cells are co-treated with TRAIL and the apoptotic cell death process is expedited.

### Caspase-8 and CFLAR are both essential for cell death initiation downstream of Golgi stress

**Figure 7:**
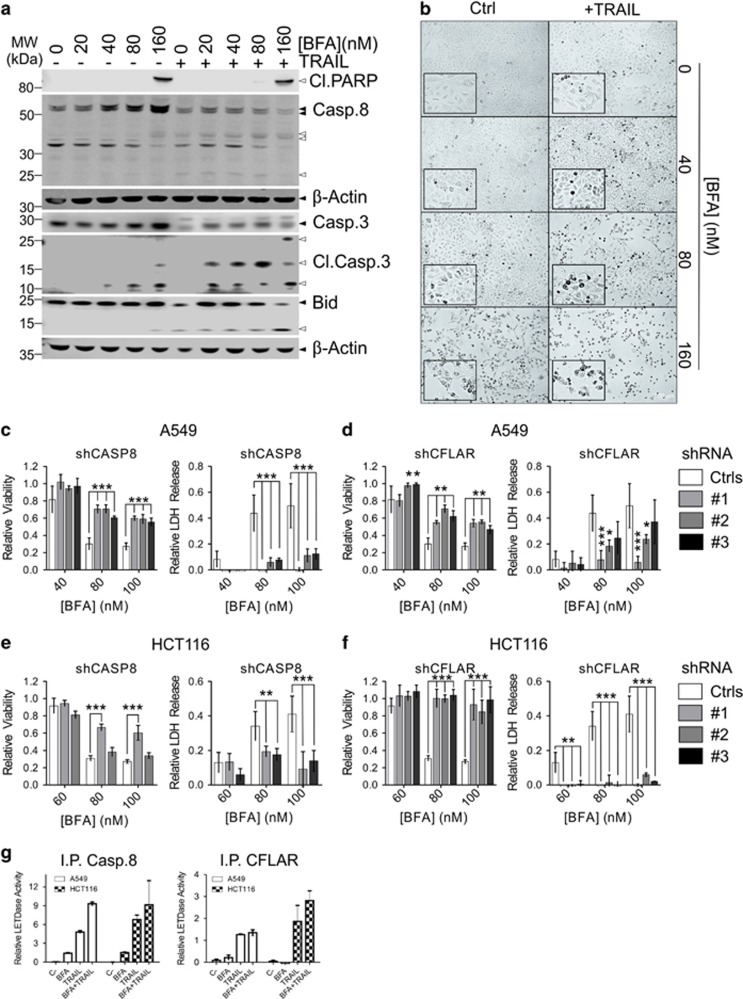
Caspase-8 and CFLAR are required for Golgi stress-induced cell death. (**a,b**) A549 cells were either left untreated or treated for 48 h with increasing concentrations of BFA alone, 50 ng/ml TRAIL or BFA in combination with TRAIL. TRAIL was added 6 h after the addition of BFA. After treatment, the cells were lysed and analyzed on western blots probed for the indicated caspases, cleaved PARP as a relative indicator of cell death, the classical caspase-8 substrate Bid and re-probed for *β*-Actin as a loading control. Black triangles indicate full-length bands of the proteins or their splice variants, white triangles their cleaved fragments, where appropriate. In addition, images were collected with a light microscope with a × 10 objective (**b**). Inserts display a further × 2 magnification of the original pictures. For LDH release data, see also Supplementary Figure S7a. (**c**–**f**) A549 (**c**,**d**) or HCT116 cells (**e**,**f**) were stably transduced with different shRNA constructs targeting either control genes (*GFP* or *Luciferase*), *CFLAR* or *caspase-8*. Caspase-8 (**c**,**e**) or CFLAR (**d**,**f**) KD cells were treated with increasing concentrations of BFA for 48 h, after which relative viability was determined with a CTB assay (left panels) and relative LDH release was determined in the cell culture supernatant as a relative measure of cell death (right panels). Relative values (treated/untreated; CTB) or ((treated/untreated)−1; LDH release) represent the mean±S.D. of three independent experiments performed in triplicate for each cell line/condition. A two-way ANOVA was performed to determine whether the phenotype of the experimental KD cells was significantly different from the pooled control KDs after treatment. *P<0.05, **P<0.01,***P<0.001. (**g**,**h**) To determine caspase-8 activation upon stimulation with BFA as compared to TRAIL or TRAIL plus BFA, A549 and HCT116 cells were stimulated for 24 h with either 100 nM BFA, 100 ng/ml TRAIL (A549 cells), 10 ng/ml TRAIL (HCT116 cells) or BFA and TRAIL, as indicated. TRAIL was added 6 h after the addition of BFA. Afterwards, the cells were collected, lysed and the lysates subjected to immunoprecipitation of either caspase-8 (left) or CFLAR (right). Relative caspase-8 activity (LETDase) on the beads was determined with a caspase-8 GLO assay. Results represent the mean±S.D. of three independent experiments performed in triplicate

DR clustering normally results in caspase-8 activation. The first step in caspase-8 activation is heterodimerization with its inactive homologue CFLAR (cFLIP_L_). In contrast to the caspase-8 homodimer, which is only formed when cells run out of CFLAR, the partially processed caspase-8 heterodimer has a restricted substrate specificity and only a weak propensity to induce apoptosis.^30, 31^ To investigate the role of caspase-8, A549 cells were stimulated with either BFA or TRAIL alone, or BFA in combination with TRAIL. TRAIL was added 6 h after the addition of BFA. Cleavage of caspase-8, caspase-3 and Bid was considerably increased in BFA/TRAIL-treated cells (Figure 7a). Upon BFA/TRAIL co-treatment, an increased number of cells display an apoptotic morphology compared to BFA-only treatment as indicated by condensed nuclei (Figure 7b and Supplementary Figure S7b). However, the rate of cell death only increased at the higher BFA levels, suggesting that an inhibitory mechanism has to be overcome before full execution of apoptosis can occur, also exemplified by the cleavage of the downstream caspase substrate PARP and the corresponding LDH release data (Figure 7a and Supplementary Figure S7a).

Caspase-8 KD and CFLAR KD A549 or HCT116 cells were resistant to BFA (Figures 7c–f and Supplementary Figures S7c and d). However, the CLFAR KD cells were still significantly sensitized to TRAIL (Supplementary Figures S7e and h), whereas the caspase-8 KD cells were resistant to TRAIL (Supplementary Figure S7f). CFLAR KD cells did not display an apparent resistance to thapsigargin (Supplementary Figure S7g). Finally, since caspase-8 cleavage is neither necessary nor sufficient for its own activation, we performed immunoprecipitation (IP) for either caspase-8 or CFLAR using lysates of A549 or HCT116 left unstimulated, stimulated with BFA, TRAIL or BFA plus TRAIL for 24 h. Afterwards, relative activity of caspase-8 (LETDase) was determined on the beads with a caspase-GLO-8 assay (Figure 7g). Very little caspase-8 activity could be detected in BFA-stimulated cells upon caspase-8 IP, and no significant activity could be detected upon IP of CFLAR. However, noticeable caspase-8 activity could be detected in TRAIL and TRAIL/BFA-stimulated cells after IP of either caspase-8 or CFLAR, suggesting the presence of both active homo- and hetero-dimers in the DISC.

## Discussion

Exploitation of ER stress and the UPR have been considered as a means to eliminate cancer cells.^32^ CHOP-mediated DR5 induction is considered an important event in cell death activation downstream of the UPR.^9, 15, 24^ Here, we demonstrate that DR4 is generally more important in the initiation of cell death downstream of Golgi stress and, in some cases, ER stress (Figure 2). In agreement with previous studies,^15, 16^ we found that cell death induction in HCT116 cells stimulated with ER stressors is mediated by DR5. However, our study shows that cell death induced by BFA or GCA in the same cellular background is mediated by DR4 (Figure 2). To our knowledge, such a distinct usage of these two highly related DRs has not previously been demonstrated.

We suggest that DR4 is initially induced in a UPR-independent manner downstream of Golgi stress, and that CHOP induction may not be essential (Figure 3). Other signaling pathways and transcription factors, such as c-Myc/MAX, probably play a role in the induction of DR4/5 as well.^10^ It is as yet unclear what would cause the DRs to accumulate in the Golgi (Figure 5). This could be due to a disruption of the vesicular traffic from the Golgi to the plasma membrane. Alternatively, lipid raft-like microdomains in the Golgi membrane could aid in the local sequestration and activation of specific signaling receptors.^33^

Induction of DR4 by Golgi stressors significantly sensitizes TRAIL-resistant cells to TRAIL (Figure 6), independent of DR4 cell surface exposure (Figure 5). We hypothesize that intracellular accumulation of DR4 upon treatment with low doses of BFA depletes freely available anti-apoptotic factors from the cell, for instance through intracellular sequestration of CFLAR. Thus, when the remaining TRAIL receptors on the cell surface are ligated, they are no longer capable of anti-apoptotic signaling and only activate caspase-8 homodimers, resulting in rapid cell death. Different TRAIL receptor agonists (TRAs) have been considered as candidates for clinical treatment of cancer.^34^ Treatment with TRAs alone has not been successful in clinical trials, but a combination of chemotherapy with TRAs seems promising. Our results suggest that specific TRAs, for either DR4 or DR5, should be used in combination with different chemotherapeutics to treat various tumors.

The two different TRAIL receptors were suggested to hetero-trimerize, and there may be some differences in the way the different trimers signal depending on their composition. For example, this might affect their propensity to activate NF-*κ*B signaling.^35^ Such a mechanism could explain the apparent discrepancy between our findings with oligomerized TRAIL and the previous findings with specific DR4/5 agonists.^29^ Early activation of constitutively expressed DR4 upon BFA treatment may induce some NF-*κ*B activation, initiating a pro-survival response.^35, 36, 37^ However, prolonged signaling induces the expression of more DR4 and limited activation of caspase-8, likely potentiated by the activation of other caspases.^38^ CFLAR-deficient cells display a reduced sensitivity to BFA (Figure 7), while no significant caspase-8 activity was associated with CLFAR in BFA-stimulated cells. This may suggest that caspase-8 has a scaffold function in Golgi-stress-induced cell death and promotes signaling, rather than being directly responsible for the initiation of apoptotic cell death, as has recently been suggested for TRAIL receptor signaling.^39^

In conclusion, we shed new light on the process of Golgi stress and demonstrate differential signaling of DR4 *versus* DR5 downstream of either Golgi or ER stress while emphasizing the function of the Golgi apparatus as a cell death initiation hub.

## Materials and methods

### Antibodies and reagents

Antibodies rabbit anti-bid (#2002), rabbit anti-cleaved PARP (Asp214) (D64E10; #5625S), rabbit anti-caspase-3 (#9662S), rabbit anti-cleaved caspase-3 (Asp175; #9661L), rabbit anti-DR4 (D9S1R; #42533S), rabbit anti-DR5 (D4E9; 8074S), rabbit anti-PERK (#D11A8), mouse anti-*β*-Actin (8H10D10, #3700S), rabbit anti-GRP78 (C50B12; #3177) and mouse anti-caspase-8 (1C12; #9746) used for IP were all from Cell Signaling Technology (Danvers, MA, USA). Mouse anti-GBF1 (#61211) from BD Transduction Laboratories (San Jose, CA, USA), mouse anti-ARF1 (sc-53168/clone 1A9/5), mouse anti-BIG1 (sc-376866), goat anti-calnexin (C-20/sc-6465) and goat anti-GM130 (P-20/sc16268) from Santa Cruz biotechnology (Dallas, TX, USA), rabbit anti-ARF4 (11673-1-AP) from Proteintech (Chicago, IL, USA). Mouse anti-FLIP mAB (7F10) for IP was obtained from Enzo Life Sciences (Farmingdale, NY, USA). All antibodies were used at a 1 : 500 dilution for western blot and 1 : 200 for flow cytometry and fluorescence microscopy.

Brefeldin A (BFA), AG1478/tyrphostin and 10058-F4 were obtained from Sigma-Aldrich (St. Louis, MO, USA), golgicide A (GCA), thapsigargin (THA) and tunicamycin (TUN) from Santa Cruz Biotechnology, monensin from Enzo Life Sciences.

Most chemicals were obtained from Sigma-Aldrich, with the exception of Nonidet P40 Substitute (Honeywell-Fluka, Morris Plains, NJ, USA), CHAPS, HePes, PIPES, MgCl_2_, EGTA (Roth, Karlsruhe, Germany) and Triton X100 (Amresco, Solon, OH, USA).

### Cell lines and cell culture

Cell lines were obtained from the ATTC/LGC Standards GmbH (Wesel, Germany) and regularly checked for mycoplasma contamination. All cell lines were maintained in high glucose (25 mM) Dulbecco’s modified Eagle’s medium (DMEM) supplemented with 2 mM L-glutamine, 200 mg/ml penicillin, 100 mg/ml streptomycin sulfate, and 10% heat inactivated fetal serum (IFS) (Gibco/Thermo Fischer, Waltham, MA, USA). Cells were cultured in humidified incubators at 37 °C and 5% CO_2_.

### Semi-quantitative real-time (RT) PCR analysis

Cells were grown in 6 cm dishes, and mRNA was isolated using the RNeasy Plus Mini kit (Qiagen, Hilden, the Netherlands). One microgram total RNA was used for the reverse transcription (RT) reaction using the Maxima First Strand cDNA Synthesis Kit (Thermo Fischer). cDNA was diluted 1 : 15 after RT for subsequent use for semi-quantitative RT PCR. QuantiNova SYBR Green master mix (Qiagen) was used and reaction volume was 25 *μ*l per Q real-time PCR reaction performed on a Rotor-Gene Q (Qiagen). Three technical replicates were run per biological replicate for calculating the mean Ct values relative to the expression of 36B4 as a reference gene.

### Virus production and generation of stable cell lines

HEK293T were seeded at a density of 800 × 10^3^ cells in 6 cm dishes 24 h before transfection. Plasmids encoding ΔVpr and pCG (VSV-G envelope protein expression vector) and 1 *μ*g of shRNA construct were transfected into HEK293T cells using 6 *μ*l of LT1 transfection reagent (Mirus Bio LLC, Madison, WI, USA). Twelve hours post-transfection, media was changed to GlutaMAX (Gibco) media plus 30% IFS. Lentiviral supernatants were collected after 48 h. Virus-containing medium was centrifuged to remove cellular debris and aliquots were frozen at −80 °C for later use.

For lentiviral shRNA transduction cells were plated at a density of 150 × 10^3^ cells in six-well plates and allowed to settle overnight. The culture medium was then replaced by 3 ml DMEM containing 10% IFS supplemented with 8 *μ*g/ml polybrene (Sigma-Aldrich), and 200 *μ*l of viral SN for A549 cells and 400 *μ*l for all other cells was added. Twenty hours later, infected cells were selected with medium containing 2 *μ*g/ml Puromycin (Gibco) and/or 350 *μ*g/ml Hygromycin (Amresco).

### Gel electrophoresis and western blot

Cells pellets were dissolved in RIPA buffer (10 mM Tris-Cl (pH 8.0), 1 mM EDTA, 0.5 mM EGTA, 1% Triton X-100, 0.1% sodium deoxycholate, 0.1% SDS,140 mM NaCl) supplemented with a complete protease inhibitor cocktail (Roche, Mannheim, Germany). After 20 min on ice, cells were sonicated for 10 s and centrifuged for 10 min at max. speed to pellet insoluble debris. Supernatants were adjusted to 400 *μ*g/ml with buffer after determining their concentration with a BCA protein assay (Pierce/Thermo Fischer). Forty micrograms was loaded per well on 4–12%, 1 mm, pre-cast NUPage gels (Invitrogen, Carlsbad, CA, USA). Protein was transferred to 0.22 *μ*m nitrocellulose membrane (Ahlstrom, Helsinki, Finland) in Tris/Glycine buffer (0.25 M Tris, 1.92 M glycine, pH approx. 8.3) supplemented with 15% EtOH (*v*/*v*; Roth) for 2.5 h at 4 °C and 300 mA. Membranes were blocked at room temperature with 5% (*v*/*v*) milk in PBS with 0.1% Tween20 (Merck, Darmstadt, Germany) before probing with primary antibodies overnight at 4 °C. The following day, membranes were washed with PBS/Tween20 and probed with secondary antibodies (donkey anti-mouse 800 CW and donkey anti-rabbit 680 RD; Li-Cor Biosciences, Bad Homburg vor der Höhe, Germany), diluted 1 : 10 000 in blocking buffer for 1 h at RT. Afterwards, membranes were washed again with PBS/Tween20, followed by a single wash with PBS and scanned on a Li-Cor Odyssey Sa infrared scanner. Blots for caspase-3 and Bid in Figure 7 were instead probed with HRP-linked secondary antibodies (Cell Signaling Technology), developed with SuperSignal West Femto substrate (Pierce) and scanned on a Li-Cor C-digit scanner.

### Viability and cell death assays

Cells were seeded at 5000 cells/well in black 96-well plates with clear bottoms, 80 *μ*l volume in DMEM medium without antibiotics. Compounds were added at 5 × concentration in 20 *μ*l medium after allowing the cells to settle overnight. After incubation with the compounds, 40 *μ*l of the culture supernatant was transferred to a clear 96-well plate to determine LDH release with the Pierce LDH cytotoxicity assay kit (Thermo Fischer Scientific), according to the manufacturer’s instructions. Relative viability was determined by adding 20 *μ*l cell titer blue reagent (Promega, Madison, WI, USA) to the cells. Living, respiring, cells convert the blue, non-fluorescent resazurin to fluorescent red resorufin. Fluorescence was determined after 3 h of incubation at 37 °C on a Glomax Multi Mode plate reader (Promega). Finally, DEVDase activity was determined by lysing the cells in caspase buffer^40^ (10 mM Pipes pH 7.2, 100 mM NaCl, 10% sucrose, 0.1% Chaps, 10 mM dithiothreitol, 1% NP-40 and 1 mM EDTA) containing 50 *μ*M of the fluorescent caspase-3/7 substrate Ac-DEVD-AFC and 100 *μ*M of the proteasome inhibitor MG132 to reduce the background signal (both from Santa Cruz Biotechnology). AFC fluorescence was measured after 2 h of incubation at 37 °C on a Glomax Multi Mode plate reader. Raw data from the CTB assay were converted into relative viability values by dividing the values from the untreated wells by the values obtained from treated wells, so that untreated values were fixed at 1. Relative LDH-release and DEVDase activity was calculated in a similar fashion, except that 1 was subtracted from the results so that the untreated values were fixed at 0 (no LDH-release/DEVDase activity).

### Flow cytometry

Cells were stimulated as described, collected by gentle scraping on ice, washed in PBS and fixed with 2% paraformaldehyde (Electron Microscopy Science, Hatfield, PA, USA) in PBS for 15 min at RT. Afterwards, cells were washed three times with PBS and either permeabilized with 0.1% Triton X100 in PBS for 15 min to detect total protein or re-suspended in PBS to detect only membrane-bound protein. The cells were washed again and blocked in 20% normal donkey serum (Jackson ImmunoResearch, Suffolk, UK) in PBS for 45 min. After blocking, the cells were re-suspended in primary antibody mix in blocking buffer and incubated overnight at 4 °C with gentle agitation to ensure maximal binding of the antibody. Finally, cells were washed again, probed for 1 h at RT with a secondary donkey anti-rabbit antibody labeled with Alexa Fluor 488 (BD Transduction Laboratories), washed, and analyzed on a BD FACS Aria IIu flow cytometer equipped with FACS Diva software.

### Fluorescence microscopy

For fluorescence microscopy, 50 × 10^3^ cells were seeded on cover slips in 24-well plates and treated for 24 h, as indicated. Cells were then washed, fixed, permeabilized and blocked on the cover slips as for flow cytometry and probed with primary antibodies overnight at 4 °C with gentle agitation. Cells were then washed again and probed for 1 h at RT with a secondary donkey anti-rabbit antibody labeled with Alexa Fluor 488 or donkey anti-goat antibody labeled with Alexa Fluor 568 (BD Transduction Laboratories). Hoechst dye (1 : 10 000; Life Technologies) was added for the last 10 min to stain the nuclei. Cover slips were then washed again and mounted with a drop of Vectashield (Vector Laboratories, Peterborough, UK) on objective slides for later analysis on a Zeiss Ax10 Observer D1 fluorescence microscope.

### Caspase-8 IP and activity assay

To determine caspase activation upon application of Golgi stress, 10^6^ A549 or HCT116 cells were seeded in 10 cm dishes and left to adhere overnight. The following day, the medium was replaced with medium containing either vehicle or BFA. TRAIL was added 6 h later. Twenty four hours after the addition of BFA, the cells were collected by gentle scraping on ice, washed with PBS and lysed in 500 *μ*l RIPA buffer supplemented with protease inhibitors. The cell suspension was then briefly sonicated, centrifuged at 14 000 r.p.m., and the cleared lysate split in two equal portions for IP. To IP caspase-8 or CLFAR, 20 *μ*l of the appropriate antibody was conjugated to 100 *μ*l washed protein G agarose beads (Invitrogen) for 2 h at 4 °C with constant rotation, followed by three washes with PBS to remove unbound antibody. The beads were then equally divided over the samples for overnight IP of either caspase-8 or CFLAR. The following day, the beads were washed again and 10 *μ*l of the beads re-suspended in PBS was used for a caspase-GLO-8 assay (Promega), performed in duplicate. Luminescence signal was read on a Glomax Multi Mode plate reader (Promega), after 1 h.

### Primers and shRNA constructs

The following primers were used for qPCR experiments: *36B4* 5′-CAGCAAGTGGGAAGGTGTAATCC-3′ (fwd), 5′-CCATTCTATCATCAACGGGTACAA-3′(rev), *TNFR1* 5′-TGCAGGAAGAACCAGTACCG-3′ (fwd), 5′-TTCGTGCACTCCAGGCTTTT-3′ (rev), *TNFR2* 5′-CATGCCGGCTCAGAGAATACT-3′ (fwd), 5′-CACCTGGTCAGAGCTACAGC-3′ (rev), *DR4* 5′-GGTCGTACCTAGCTCAGCTG-3′ (fwd), 5′-CTGTACATGGGAGGCAAGCA-3′ (rev), *DR5* 5′-GTGGAGCTAAGTCCCTGCAC-3′ (fwd), 5′-TCCCCACTGTGCTTTGTACC-3′ (rev), *CD95* 5′-CCATAAGCCCTGTCCTCCAG-3′ (fwd), 5′- TGGTATTCTGGGTCCGGGT-3′ (rev), *CHOP* 5′-CATCACCACACCTGAAAGCA-3′ (fwd), 5′-TCAGCTGCCATCTCTGCA-3′ (rev), *CFLAR* 5′-GAACAGCTTGGCGCTCAAC-3′ (fwd), 5′-GCCAAGAATCTGGGATATACCATG-3′ (rev). The results were normalized to 36B4 expression using the 2^−ΔΔCt^ method.^41^ Primers were designed with Geneious 10.0.5 software (Biomatters Ltd, Auckland, New Zealand) and synthesized by Eurofins Genomics (Ebersberg, Germany).

The following shRNA constructs were obtained from Sigma for specific knockdown of the indicated target genes:


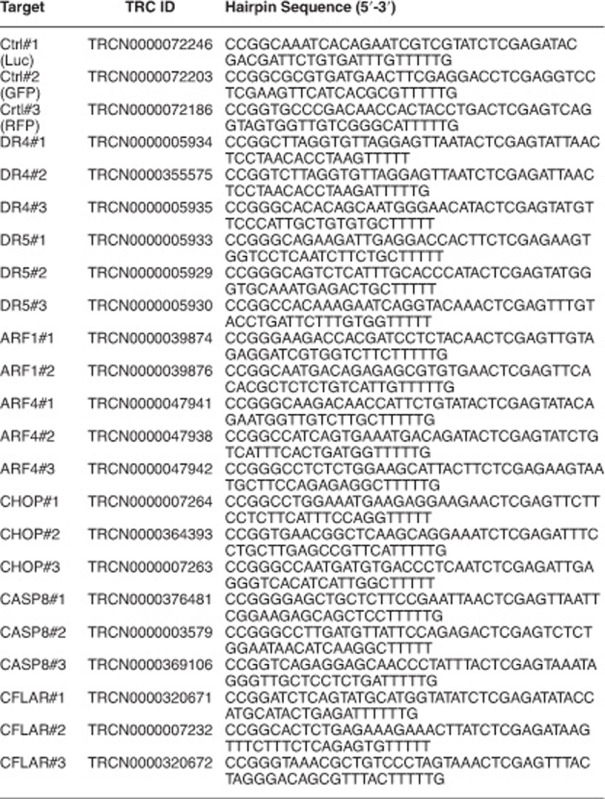


### Software and statistical analysis

Statistical analysis was performed with and graphs were drawn in GraphPad Prism 5 (GraphPad Software Inc., San Diego, USA). One-way or two-way ANOVA with Bonferroni’s multiple comparison tests were performed to determine significant differences between groups, as indicated. *P*-values <0.05 were considered significant. Western blot data were analyzed in Image Studio 3.1.4 (Li-Cor). Flow cytometry data were analyzed with FlowJo V10 (FlowJo, Ashland, OR, USA). Fluorescence micrographs were obtained and analyzed with Zen 2.3 software (Zeiss, Oberkochen, Germany). The online search engine TFBind (http://tfbind.hgc.jp/) was used to identify transcription factor binding sites in the *DR4*, *DR5* and *ARF4* promotor region up to 1000 bp upstream of the first transcription start codon. Other data were processed in Microsoft Excel 2013 (Microsoft Inc., Redmond, WA, USA).

## Publisher's Note:

Springer Nature remains neutral with regard to jurisdictional claims in published maps and institutional affiliations.
