# Supplementary material for: Secretory stressors induce intracellular death receptor accumulation to control apoptosis
Source: Cell Death Dis. 2017 Oct 5;8(10):e3069–. doi: 10.1038/cddis.2017.466 (PMC5680588; doi:10.1038/cddis.2017.466)
Supplement: Supplementary Figure Legends [file cddis2017466x1.docx]

**Supplemental Figure Legends**

**Figure S1: Induction of Death Receptor 4 and 5 upon secretory stress induction**

**a:** BCPAP cells were incubated with vehicle (EtOH), BFA (100 nM), GCA (1 µM), MNS (5 µM) or tunicamycin (TUN; 10 µM) for 24 h after which mRNA was isolated and cDNA prepared. The cDNA was used to determine the relative expression of the indicated TNF-receptor superfamily members by RT-PCR. The fold change relative to the vehicle treated control is shown. Data represent the mean ±SD of duplicate experiments.

**b-c:** A549 cells were either incubated with vehicle (EtOH), tyrphostin (2 μM) or thapsigargin (2 μM) for 24 h, after which mRNA was isolated and cDNA prepared to determine relative *DR4* (**b**) and *DR5* (**c**) expression by qPCR. Data represent the mean ±SD of three independent experiments with two technical replicates each. A two-way ANOVA was performed to determine whether the expression levels found in the treated samples were significantly different from the vehicle controls. ** *P*<0.01,*** *P*<0.001 (two-way ANOVA).

**Figure S2: Knock-down of DR4 protects cells from Golgi-stress-induced cell death**

**a-b:** Dose response curves of various DKD cell lines as in Figure 2 showing the response to BFA (left) and thapsigargin (THA; right) titrated on A549 (**a**) or HCT116 (**b**) cells displaying the log inhibitor concentration plotted against relative viability as determined with a CTB assay. EC50 values ± SD are tabulated on the right.

**c:** HeLa cells were stably transduced with different shRNA constructs targeting either control genes (Ctrl; GFP or RFP), DR4 or DR5. Relative *DR4* (left) and *DR5* (right) mRNA levels were determined by qPCR. Data represent the mean ±SD of triplicate samples.

**d:** DR4 protein levels of HeLa cells stably transduced with different shRNA constructs targeting either control genes, *DR4* or *DR5* were determined on Western blot probed with a specific antibody against DR4 or against HSP90 as a loading control. The dotted line indicates where the blot was cropped to excise two redundant lanes. Black arrowheads indicate the full-length proteins, the white arrowhead indicates the glycosylated form of DR4.

**e-f:** To determine the sensitivity of DR4 (**e**) and DR5 KD HeLa cells KD (**f**) to Golgi stress, the cells were treated with increasing concentrations of BFA (left), GCA (middle) or monensin (MNS; right) for 48 h, after which relative viability was determined with a CTB assay. Relative values (treated/untreated) represent the mean ±SD of triplicate experiments.

**Figure S3: Inhibition of c-Myc/MAX prevents induction of DR4/5 upon BFA treatment**

A549 cells were treated with 100 nM BFA or left untreated in the absence or presence of 50 µM of the c-Myc/MAX inhibitor 10058-F4. Samples were taken at the indicated time points for Western blot analysis (**a**) or to determine LDH release in the cell culture supernatant as a relative measure of cell death (**b**). Blots were probed with specific antibodies directed against the indicated proteins. Black arrowheads indicate the full-length proteins and their splice variants, white arrowheads their cleavage products. The blots were re-probed for β-Actin as a loading control. Representative LDH release data ((treated/untreated)-1) is shown as the mean ±SD of triplicate samples.

**Figure S4: ARF4 knock-down** **protects from Golgi-stress-induced cell death and induction of DR4/5**

**a-b:** HeLa cells stably transduced with different shRNA constructs targeting either a control gene (Ctrl; *RFP*), *TRAPPC11, TRAPPC12, TRAPPC13, ARF1* or *ARF4* were incubated for 48 h with vehicle (EtOH, 0) or increasing concentrations of BFA (**a**) or tunicamycin (TUN; **b**). Afterwards, lysates were prepared for Western blot and probed with specific antibodies directed against the indicated proteins and re-probed with an antibody against β-Actin as a loading control. Black arrowheads indicate full-length proteins and their splice variants, white arrowheads their cleavage products or modified species of the proteins. Blots are representative of three independent experiments.

**Figure S5: Enlargement of Figure 5c-d**

**a-b**: A549 cells were grown on microscopy cover slips and incubated with either vehicle (EtOH), 100 nM BFA or 100 nM thapsigargin (THA) for 24 h. Afterwards, the cells were washed, fixed, permeabilized and probed overnight with specific antibodies against either the ER marker Calnexin (CNX; **a**) or the Golgi marker GM130 (**b**) in combination with an antibody against DR4. The following day, cells were washed again and probed with fluorescently labeled secondary antibodies to detect the localization of DR4 as well as Hoechst to stain the nuclei. Coverslips were then mounted on slides and analyzed by fluorescence microscopy. Representative images of single channels and overlays are shown.

**Figure S6: Induced expression of DR4 by Golgi/ER stressors sensitizes A549 cells to TRAIL**

**a-b:** HCT116 cells stably transduced with shRNA constructs targeting either control genes (*Luciferase* or *GFP*), *DR4, DR5* or both were incubated with increasing doses of TRAIL for 48 h. Afterwards, relative cell viability was determined with a CTB assay (**a**) while LDH release in the cell culture supernatant was used as a relative measure of cell death (**b**). Relative values (treated/untreated; CTB) or ((treated/untreated)-1; LDH release) represent the mean ±SD of three independent experiments performed in triplicate for each cell line/condition. A two-way ANOVA was performed to determine whether the phenotype of the experimental KD cells was significantly different from the pooled control KDs after treatment. * *P*<0.05, ** *P*<0.01,*** *P*<0.001.

**c-f:** A549 cells were incubated with increasing concentrations of GCA (**c**), thapsigargin (THA; **d**), monensin (MNS; **e**), tunicamycin (TUN; **f**) or AG1478 (tyrphostin; **g**) in the presence (black bars) or absence (white bars) of 25 ng/mL TRAIL for either 24 h (top) or 48 h (bottom). TRAIL was added 6 hours after the addition of the other compounds or medium. Afterwards, relative cell viability was determined with a CTB assay (right) while LDH release in the cell culture supernatant was determined as a relative measure of cell death (left). Relative values (treated/untreated; CTB) or ((treated/untreated)-1; LDH release) represent the mean ±SD of triplicate experiments.

**Figure S7: Caspase-8 and CFLAR are required for Golgi stress-induced cell death**

**a:** LDH-release data associated with Figure 7a. Cell culture supernatant samples were taken after a 48 treatment duration with increasing concentrations of BFA to determine LDH release as a relative measure of cell death. Relative values ((treated/untreated)-1) represent the mean ±SD of triplicate samples. Results are representative of at least three independent experiments.

**b:** Enlarged images of Figure 7b. A549 cells were either left untreated or treated for 48 h with increasing concentrations of BFA alone, 50 ng/mL TRAIL or BFA and TRAIL in combination. TRAIL was added 6 hours after the addition of BFA. Images were collected with a light microscope with a 10x objective.

**c:** Lysates of A549 and HCT116 CASP8 (C8) or control (Ctrl) KD cells were prepared (see **Figure 7c and e**) and analyzed on Western blot probed with specific antibodies against caspase-8 and re-probed for β-Actin as a loading control to determine KD of caspase-8. The dotted line indicates where the blot was cropped to remove redundant lanes.

**d:** CFLAR KD in A549 (left) and HCT116 cells (right) stably transduced with different shRNA constructs targeting either *CFLAR*  or control (Ctrl) genes (*Luciferase* or *GFP*) (see **Figure 7d and f**) was determined by qPCR. Data represent the mean ±SD of three technical replicates. A one way ANOVA was performed to determine whether *CFLAR* mRNA levels were significantly reduced in the experimental KD as compared to the pooled controls. *** *P*<0.001.

**e-h**: CFLAR or CASP8 KD or control cells were treated for 48 h with increasing concentrations of TRAIL (**e**, **f** and **h**) or thapsigargin (THA; **g**), after which relative viability was determined with a CTB assay (left panels) and relative LDH release was determined in the cell culture supernatant as a relative measure of cell death (right panels). Relative values (treated/untreated; CTB) or ((treated/untreated)-1; LDH release) represent the mean ±SD of three independent experiments performed in triplicate for each cell line/condition. A two-way ANOVA was performed to determine whether the phenotype of the experimental KD cells was significantly different from the pooled control KDs after treatment. * *P*<0.05, ** *P*<0.01,*** *P*<0.001.
