# Supplementary figures and images for: Secretory stressors induce intracellular death receptor accumulation to control apoptosis
Source: Cell Death Dis. 2017 Oct 5;8(10):e3069–. doi: 10.1038/cddis.2017.466 (PMC5680588; doi:10.1038/cddis.2017.466)

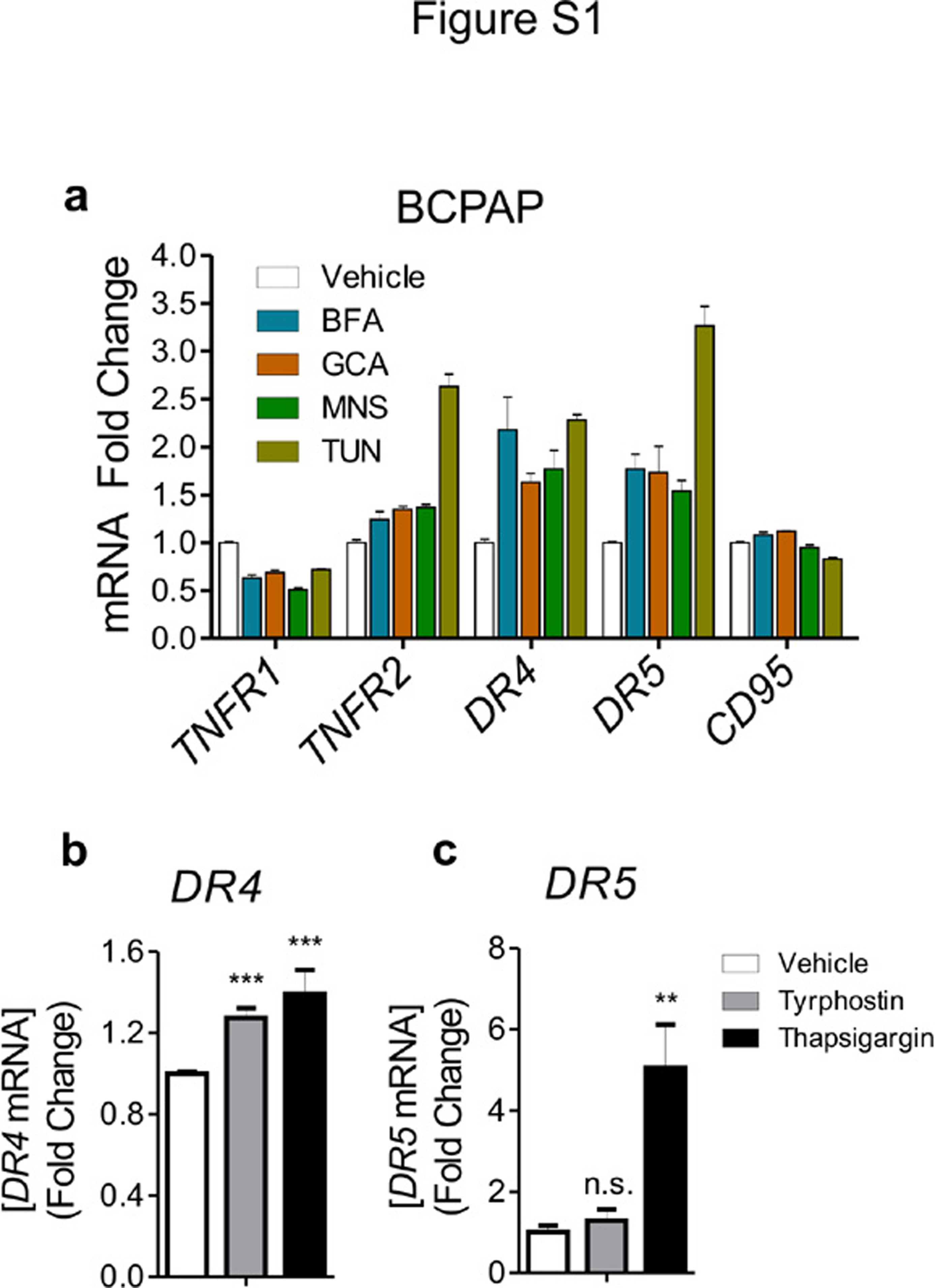

Supplement: Supplementary Figure 1 [file cddis2017466x2.tif]

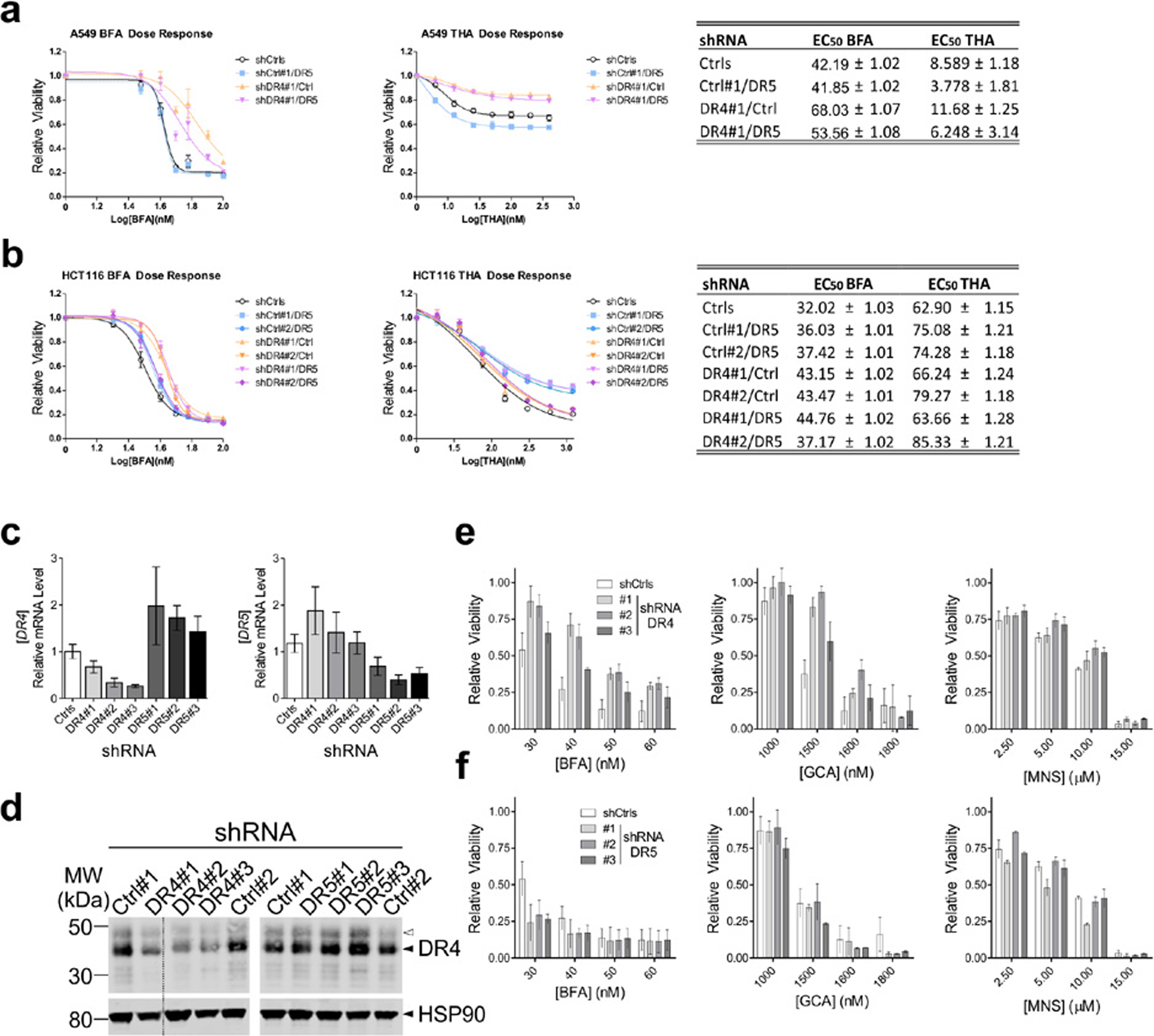

Supplement: Supplementary Figure 2 [file cddis2017466x3.tif]

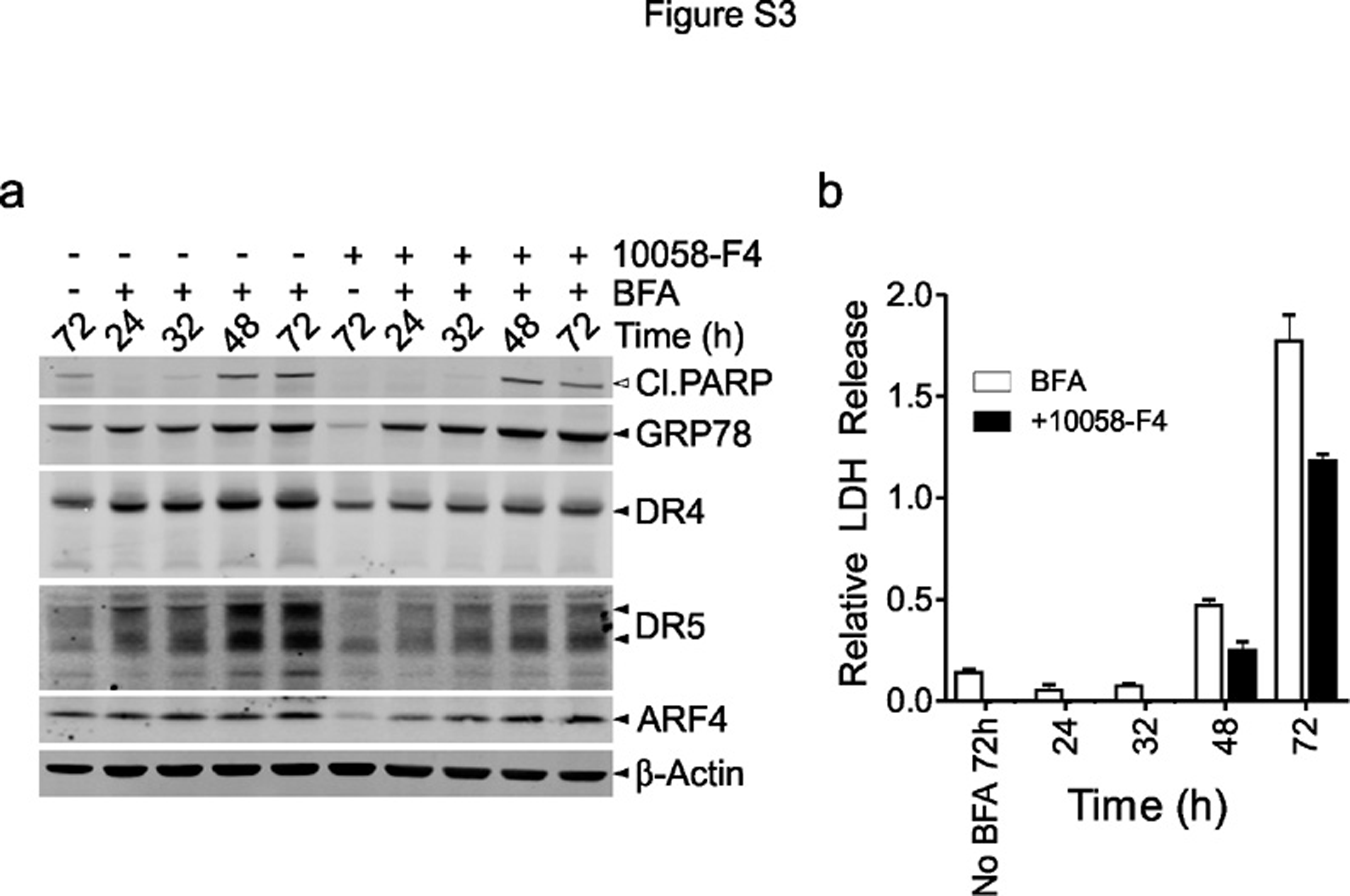

Supplement: Supplementary Figure 3 [file cddis2017466x4.tif]

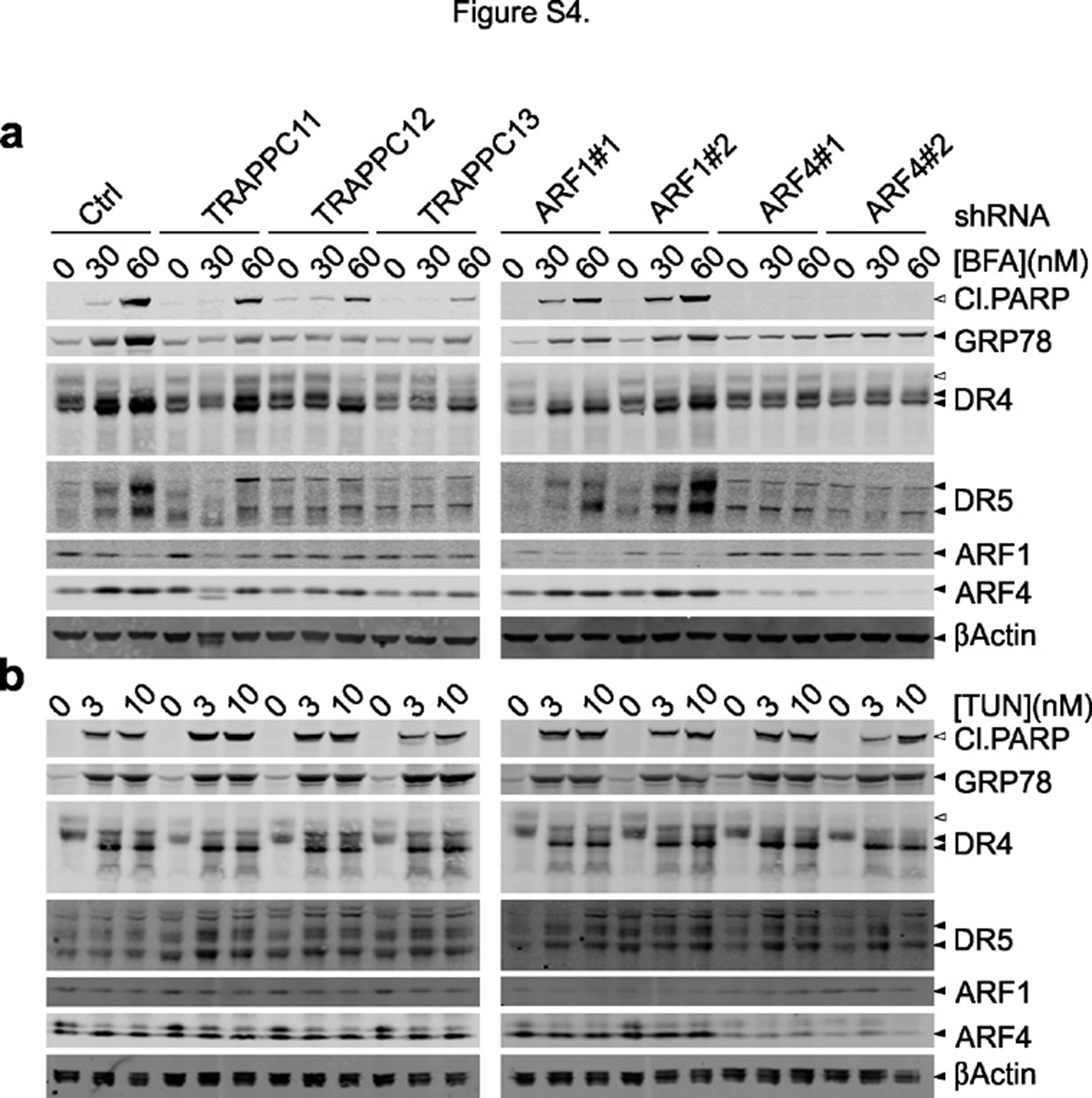

Supplement: Supplementary Figure 4 [file cddis2017466x5.tif]

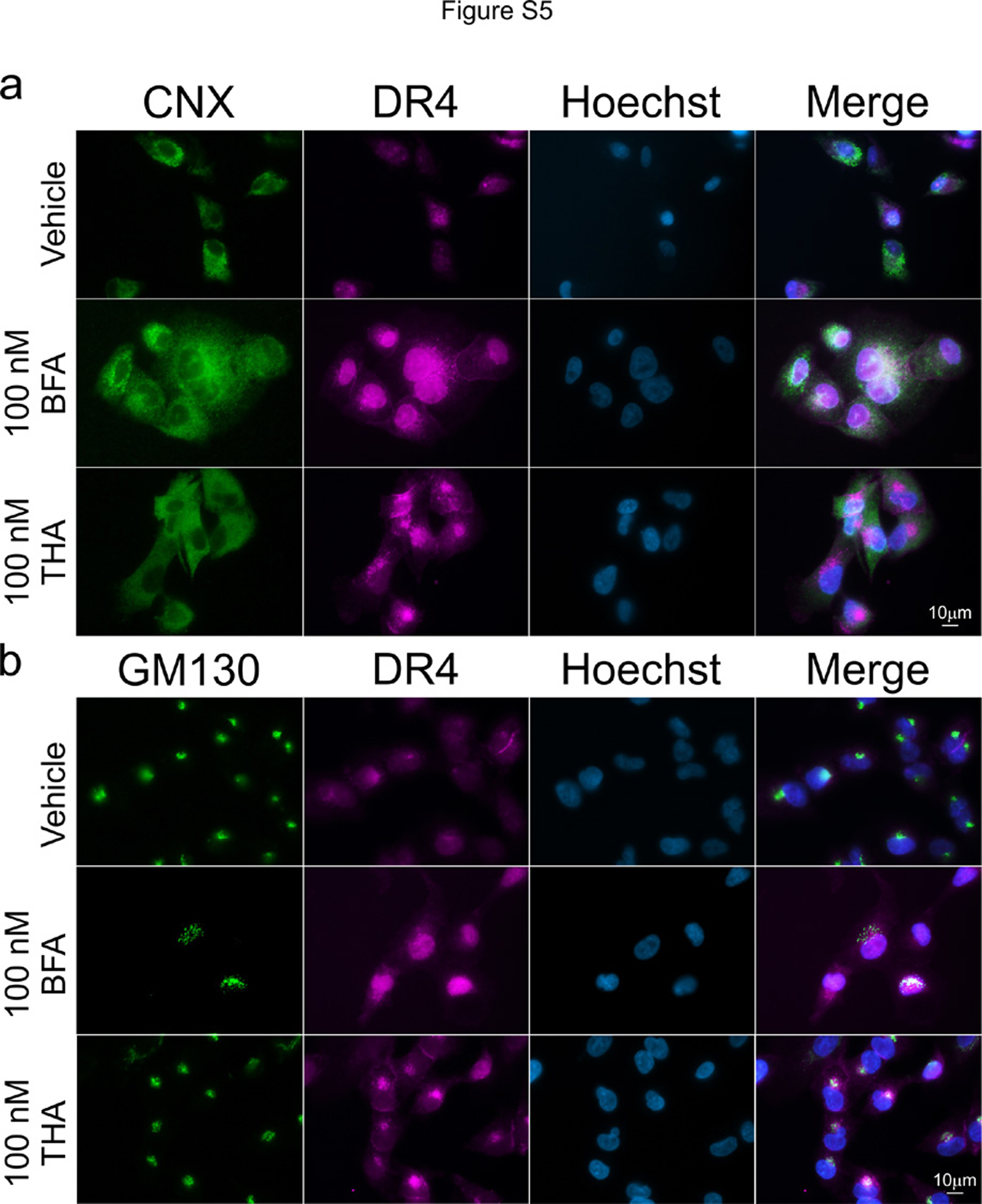

Supplement: Supplementary Figure 5 [file cddis2017466x6.tif]

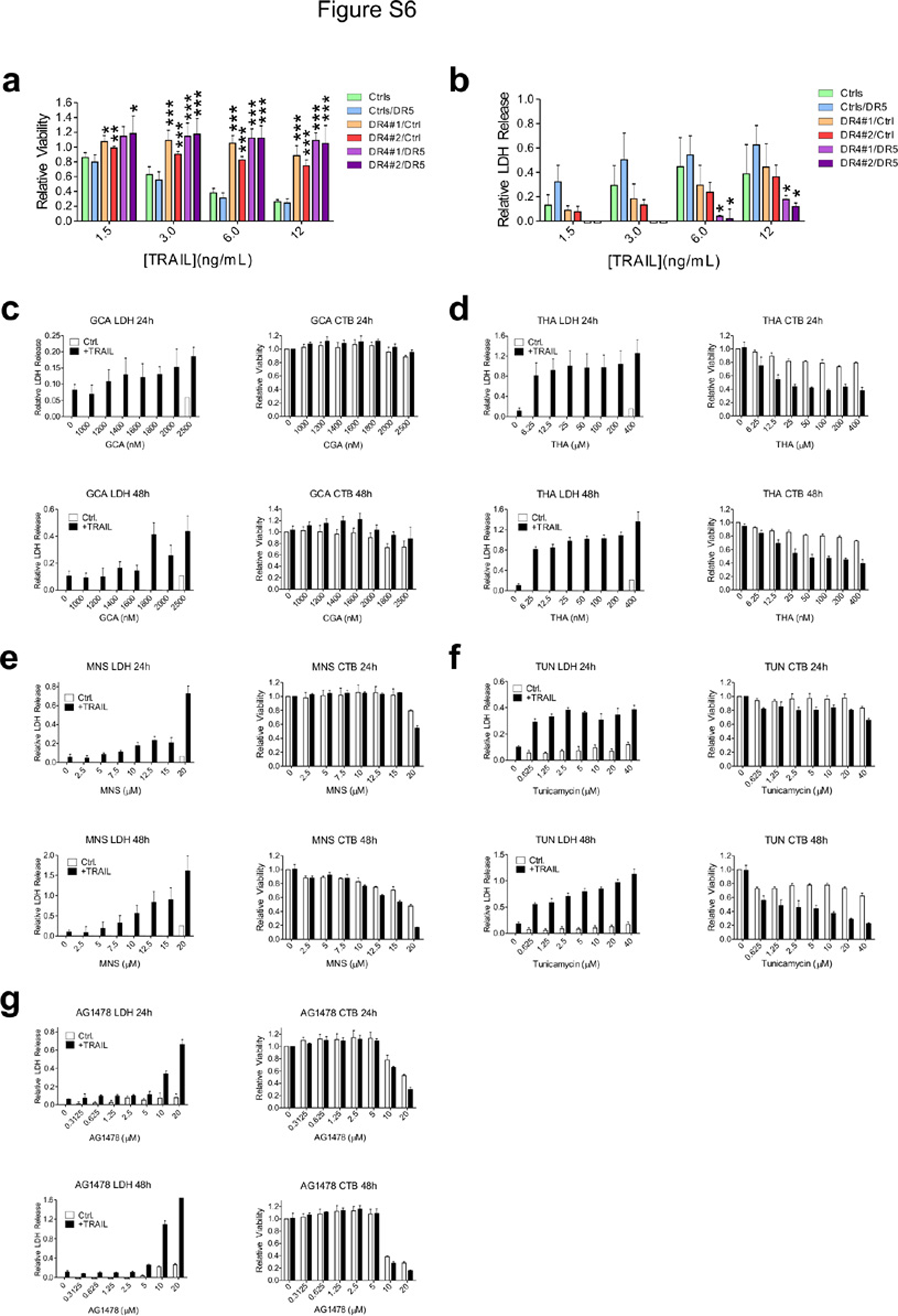

Supplement: Supplementary Figure 6 [file cddis2017466x7.tif]

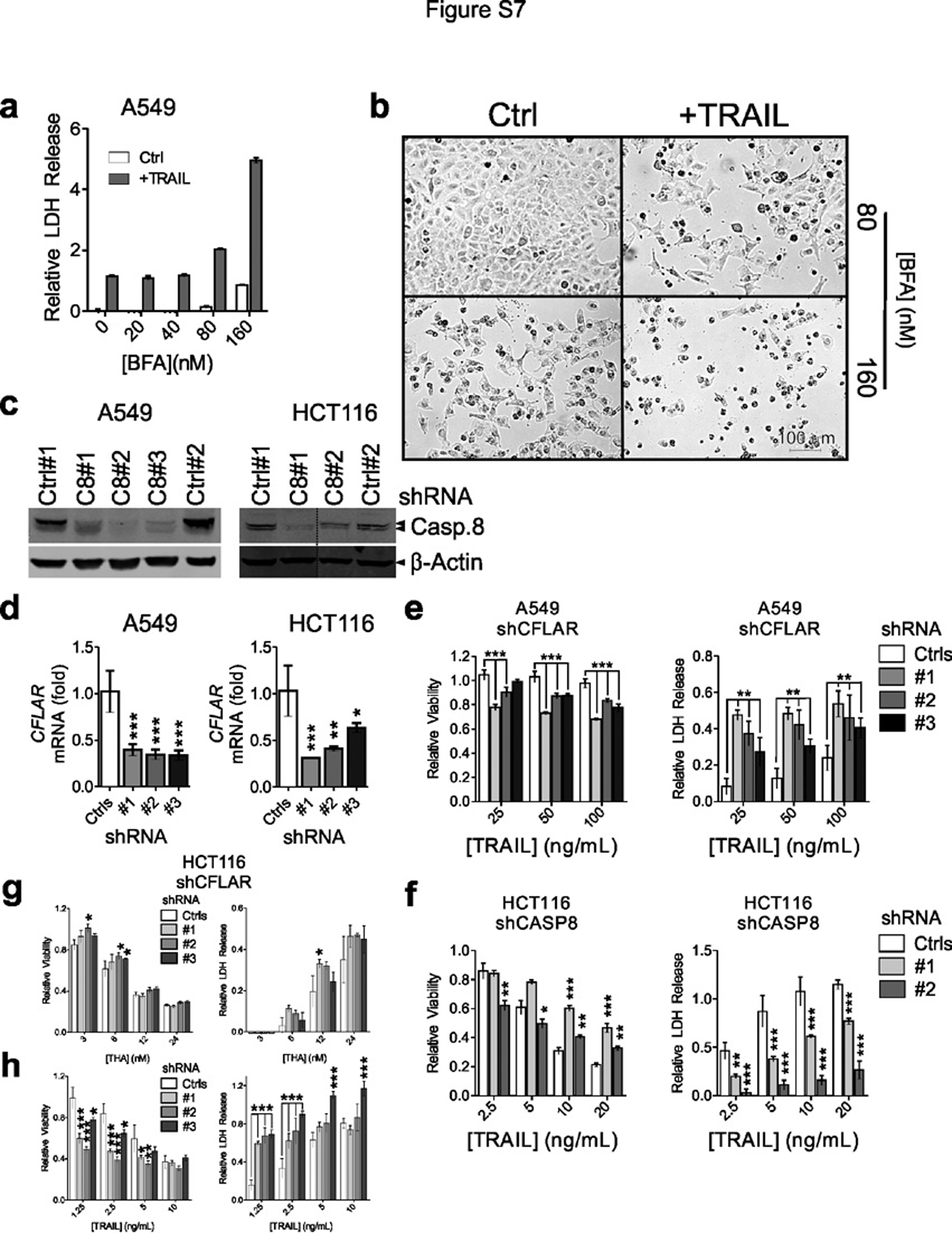

Supplement: Supplementary Figure 7 [file cddis2017466x8.tif]
